# Fellowship of the European Board of Surgery in the specialty of Minimally Invasive Surgery (F.E.B.S./MIS): a continuous evaluation

**DOI:** 10.1007/s00464-025-12204-3

**Published:** 2025-09-19

**Authors:** Adisa Poljo, Jennifer M. Klasen, Nathan J. Curtis, Marek Soltes, Nader K. Francis, Dorin Popa, Milos Bjelovic, Predrag Andrejevic, Beat P. Müller, Felix Nickel, Georg Bischof, Lars Fischer

**Affiliations:** 1 Department of Visceral Surgery, Clarunis University Digestive Health Care Center Basel, St. Clara Hospital and University Hospital Basel, Basel, Switzerland; 2 Medical Faculty, Johannes Kepler University Linz, Linz, Austria; 3 Department of Surgery, Dorset County Hospital NHS Foundation Trust, Dorchester, UK; 4 1st Department of Surgery, Pavol Jozef Safarik University in Kosice, Tr. SNP 1, 040 01 Kosice, Slovak Republic; 5 The Griffin Institute, The Northwick Park Institute for Medical Research, St Marks Hospital, London, UK; 6 Department of Surgery, Yeovil District Hospital, Yeovil, UK; 7 Department of Surgical Biotechnology, University College London, London, UK; 8 Department of Surgery, Linköping University Hospital, Linköping, Sweden; 9 Euromedik General Hospital, Belgrade, Serbia; 10 School of Medicine, University of Belgrade, Belgrade, Serbia; 11 Department of Surgery, Mater Dei Hospital, L-Imsida, Malta; 12 Department of General, Visceral and Thoracic Surgery, University Medical Center Hamburg-Eppendorf, Hamburg, Germany; 13 Department of General and Vascular Surgery, Evangelic Hospital, Vienna, Austria; 14 Department of General, Visceral and Metabolic Surgery, Klinikum Mittelbaden, Baden-Baden, Germany

**Keywords:** Minimally invasive surgery, Surgical education, Examination, Board certification

## Abstract

**Background:**

Minimally Invasive Surgery (MIS) has become the standard approach for many procedures, driving rapid changes in training pathways and challenging traditional assessment and accreditation methods. To address this, the European Union of Medical Specialists (UEMS), in collaboration with the European Association for Endoscopic Surgery (EAES), established a working group in 2015 to develop a MIS-specific board fellowship exam (Fellow of European Board of Surgery in Minimally Invasive Surgery (F.E.B.S./MIS)). This rigorous, multi-modality examination assesses surgeons’ knowledge and skills to ensure high-quality independent practice. This study provides an overview of the exam’s development, structure, and quality assurance, with a focus on participant evaluation.

**Methods:**

Eligibility followed UEMS criteria, including certified MIS training, case logbook documentation, and English proficiency. The exam comprised a 100-item multiple-choice test (MCQ) and an objective structured clinical examination (OSCE) with clinical scenarios and validated technical skill tasks. Participants completed evaluation questionnaires on exam experience. Data were analyzed using descriptive statistics, linear regression, and independent-samples t-tests to examine associations between experience, performance, and total scores.

**Results:**

Between 2018 and 2024, 119 participants from 28 countries undertook the exam in seven European countries. Most were experienced attending surgeons, with pass rates of 61–88%. Higher credit scores were linked to passing, though not directly correlated, indicating experience alone did not ensure success. Fellowships were considered as the optimal exam time, with motivations including certification and knowledge updates. Feedback was highly positive, especially for oral case-based stations, and nearly all recommended the exam. Suggested improvements included streamlining the application process, enhancing practical training opportunities, offering flexible dates, and enabling exams in candidates’ home countries or languages.

**Conclusion:**

The UEMS/EAES MIS Board exam is firmly established as a specialized certification for MIS and has been well received by participants. Nevertheless, its broader influence and professional recognition still require systematic assessment.

**Graphical abstract:**

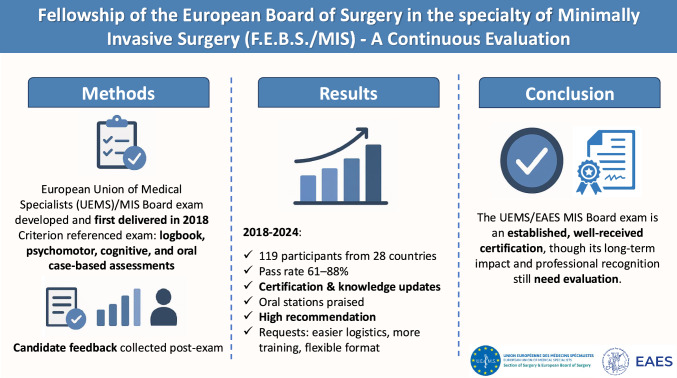

**Supplementary Information:**

The online version contains supplementary material available at 10.1007/s00464-025-12204-3.

## Background

Minimally invasive surgery (MIS) has gained widespread acceptance, becoming the standard approach globally for many surgical procedures [1]. The integral role of laparoscopy in multidisciplinary practice has led to marked shift away from open surgery due to the associated patient and healthcare provider benefits seen with MIS techniques. This has impacted training experiences and needs, as highlighted by a 462% increase in laparoscopic procedures performed by residents between 2000 and 2018 [2]. While many training systems have successfully adapted to ensure safe acquisition and delivery of MIS skills, the assessment mechanisms for trainees have arguably lagged behind with potential critical gaps in formalized training assessment and certification.

The European Union of Medical Specialists (UEMS) organizes high-level examinations for the European Specialist Qualification (EBSQ) award [3, 4]. This achievement acknowledges the knowledge, skills, and professionalism of surgeons at the European level. It has become a quality mark and is increasingly prestigious among professionals and institutions. The UEMS recognized that the pace of MIS expansion across European nations meant existing examination categories may not sufficiently assess routinely performed MIS procedures and skill sets. In 2015, a new working group composed of renowned university-affiliated European MIS specialists was commissioned by the UEMS Section of Surgery to design, develop, and deliver a new MIS-specific EBSQ examination with completion leading to the award of the title "Fellow of the European Board of Surgery/MIS" (F.E.B.S./MIS). The working group is a formal collaboration with the European Association for Endoscopic Surgery (EAES) with a memorandum of understanding signed by the active presidents of both societies. By aligning with UEMS principles of training standardization, fostering mutual recognition of qualifications, and facilitating professional mobility, the F.E.B.S./MIS aims to ensure candidates have displayed sufficient MIS competencies and are well-equipped to deliver high-quality independent practice and to advance cross-border collaboration among surgeons [4]. From the outset, the working group incorporated quality assurance assessments including participant feedback to shape continuous improvement.

This report aims to provide an overview of the exam development, structure, and quality assurance of its delivery. Focus was set on participant and logbook evaluation to assess the relevance, comprehensiveness, and acceptability of the exam, while enhancing transparency for future candidates.

## Methods

### UEMS MIS board exam eligibility

The exam adopts established UEMS requirements for an initial formal eligibility check followed by a multi-modality assessment process. To be eligible, candidates must meet specific UEMS stated criteria. First, they should be trained in one of the 27 European Union countries or a UEMS affiliated nation (Iceland, Norway, Switzerland), an associated UEMS country or a country with UEMS observer status. Those trained outside the UEMS area may also be eligible if their training and qualifications are deemed equivalent by the Eligibility Committee. Additionally, candidates must be proficient in English, with exams potentially available in other languages at the executive’s discretion and upon identification of demand.

A national Certificate of Completion of Specialist Training (CCST) is mandatory for eligibility. Candidates must also demonstrate experience in MIS, including documented case numbers for "Knowledge and Skills". A signed logbook with the corresponding credit scores meeting UEMS criteria must be submitted for assessment by two independent assessors. The logbook tracks participants’ surgical and educational activities across four categories, with a total requirement of 1000 points. Category A, for surgeons performing endoscopy, records endoscopic cases. Category B covers basic laparoscopic operations, while Category C includes advanced laparoscopic procedures based on complexity and involvement. Category D records CME credits and hands-on training. A detailed version, specifying requirements with and without endoscopy, is provided in the amendments (Supp. Material Fig. S1a and S1b). Flexibility in subspecialty case mix is permitted to reflect differing practices but core conditions and procedures are mandatory. Reflecting differing European practices, candidates may choose between a logbook with or without endoscopy. Those not performing endoscopy are not penalized. Full details and indicative numbers are provided on the examination website [4].

### UEMS MIS examination

Independent surgical practice requires a broad range of knowledge and skills. The exam was therefore designed to consist of three distinct parts delivered once per year. First, a multiple-choice test (MCQ) mapped to all areas of the curriculum with single best answer questions from five options with no negative marking is conducted. A large question bank was developed by the examination board and members of the EAES education & training committee, supplemented by senior European surgeons with examination experience invited to participate in question-writing and piloting. Each edition of the MCQ exam comprises a new set of 100 questions. In response to the pandemic, since 2020 the MCQ test is delivered online under strict exam conditions using a dedicated platform and UEMS examiners who are invigilating. The second part is an objective structured clinical examination (OSCE) circuit comprising of two core components: three MIS clinical cases with structured oral questioning being performed, and a practical MIS skills circuit using three validated tasks delivered on simulators usually during the annual EAES congress. The OSCE assessment is designed to evaluate candidates through a circuit of six stations, each lasting 10 min, for a total duration of 60 min. During the circuit, candidates rotate through all stations, where they are assessed by examiners on both their interpretive and technical abilities.

Each station presents a different clinical scenario or task. Candidates may be required to interpret a wide range of diagnostic materials, including laboratory investigations, X-rays, CT scans, MRIs, ultrasound images, and clinical photographs. Depending on the station, they may also be asked to provide oral or written responses to support their interpretations.

**Fig. 1:**
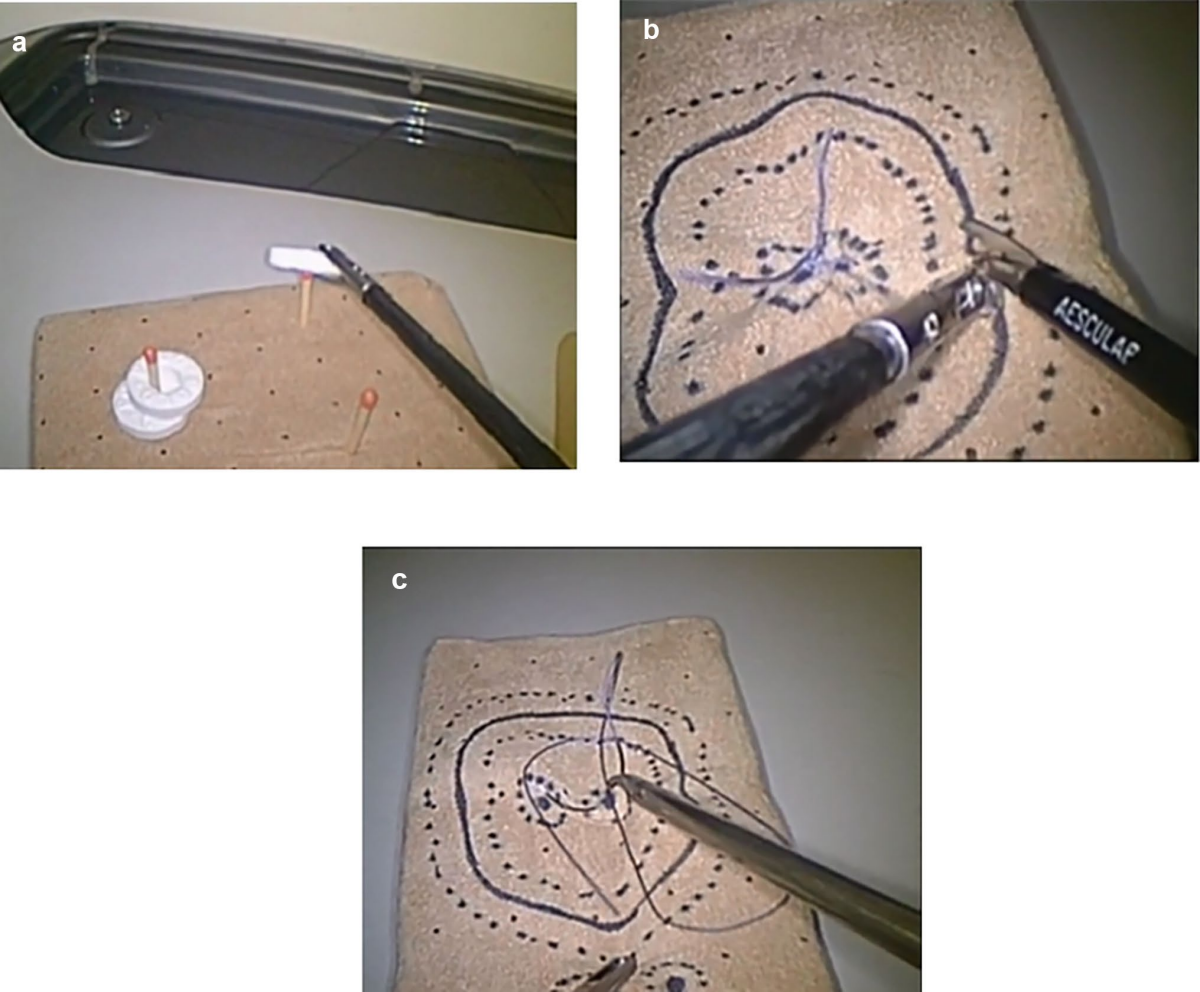
Technical skills assessment tasks. **a** Task 1: Bimanual dexterity – Basic grasping and manipulation, **b** Task 2: Dissection exercise – cutting a predefined shape, **c** Task 3: Suturing skills – suturing and knot tying

In addition to interpretive tasks, three of the stations are specifically focused on technical skills. These include assessments of bimanual dexterity, dissection, and suturing. Task 1 involves basic grasping and manipulation exercises using both hands (Fig. 1a). Task 2 is a dissection task that requires cutting a predefined shape with precision (Fig. 1b). Task 3 focuses on suturing skills, including accurate needle handling and secure knot tying (Fig. 1c). The detailed methodology and structure of these technical tasks are described and validated in the LapPass® paper, which serves as the reference standard for task execution and assessment criteria [5].

Each station is staffed by two independent examiners, resulting in a total of 12 examiners across the full circuit. All examiners are EAES committee members, selected from a pool of individuals without conflicting commitments during the exam period. For this iteration of the OSCE, members of the EAES committees will also observe the process for the first time, as part of the ongoing development and validation of the assessment format.

The total score available is 600 points: 300 from the MCQ and 300 from the OSCE assessments. Similar to other UEMS exams, the pass mark is criterion-referenced and adjusted annually based on observed scores, but typically candidates must achieve at least 400/600 (66%) points and pass all three aspects of the exam. Results are given face to face with individual feedback shortly after completion of the skills assessment. Successful candidates are invited to receive their certificate on stage at the EAES General Assembly.

### Evaluation questionnaire

All F.E.B.S./MIS examination participants were required to complete an evaluation questionnaire upon completion of the final tests prior to the release of the results (Supp. Material Figure S2). Participant data was collected, including how they first learned about the exam, as well as their motivations for undertaking it and their experience. Additionally, their level of clinical MIS experience and their views on the most appropriate stage in a surgeon’s career to take the exam were evaluated. During the COVID-19 pandemic, exam delivery faced significant challenges due to lockdowns, restrictions on in-person congress attendance, and disruptions in normal operations. Exam delivery continued but as a result of the disruption, for some analyses participant data from 2020 to 2022 was grouped together. Participants were also asked about their general impressions of the examination, including its structure and execution. A specific focus was placed on the practical component, assessing whether candidates felt the adopted tasks accurately reflected the skills necessary for performing MIS. Furthermore, the survey evaluated the perceived usefulness of various examination components in the participants’ daily clinical practice. In some questions, participants were asked to assign grades on a Likert scale from 1 (best) to 5 (worst) and free text areas were included. Other key questions included whether participants would choose to take the exam again and whether they would recommend it to colleagues. The survey concluded by inviting participants to offer recommendations for improving the examination process and asking them if they would prefer to take the MCQ as an “open book” exam. After completion of the exam the entire group had the opportunity to give oral feedback to the examiners as a group and at the time of receiving their results.

### Statistical analysis

Linear regression analyses were performed to assess associations between the number of laparoscopic operations performed and OSCE scores, MCQ points and CME points, and between participants’ total score and their total credit score. The F-statistic was used to evaluate the overall significance of each regression model. Differences in MCQ, CME, and total credit scores between participants who passed and those who failed the exam were assessed using independent-samples t-tests. A p-value of < 0.05 was considered statistically significant. Participant and exam data is presented as descriptives and means with standard deviations unless otherwise stated.

## Results

### Participants

Between 2018 and 2024, 119 participants took the F.E.B.S./MIS exam (Table 1). Of these, 75 (63%) submitted the evaluation sheet. Participants were from 28 countries across five continents. Participants reported a mean of 11.5 ± 4.3 years of clinical experience and 1202 ± 1228 MIS procedures at the time of application. This matched the data submitted for eligibility assessment. Overall, most participants taking the exam were attending surgeons (69%) followed by fellows (16%), and chiefs of units (11%). Specialists shortly after completion of residency comprised the smallest group, making up only 4.2% (Table 2).

**Table 1:** Baseline characteristics

	2018	2019	2020–2022	2023	2024	Overall
Participants (*n*)	7	17	57	22	16	119
Responses (*n*; %)	7 (100.0)	17 (100.0)	16 (28.1)	22 (100.0)	13 (81.3)	75 (63.0)
Clinical experience (years)	11.6 ± 2.4	11.0 ± 4.1	10.3 ± 3.9	11.6 ± 7.1	12.9 ± 3.4	11.5 ± 4.3
MIS cases performed* (*n*)	1057 ± 943	1018 ± 548	798 ± 254	1916 ± 1130	1219 ± 1544	1202 ± 1228

Values are presented as mean ± standard deviation; COVID-19 pandemic implications in Europe reduced feedback responses and so data from 2020 to 2022 was pooled. *at the time of registering for the exam

**Table 2:** Participant reported position at the time of taking the exam

	2018	2019	2020–2022	2023	2024	Overall
n	7	17	16	20	13	73
Specialist (n; %)	2 (28.6)	0	0	1 (5.0)	0	3 (4.1)
Fellow (n; %)	0	4 (23.5)	2 (12.5)	3 (15.0)	3 (23.1)	12 (16.4)
Attending/consultant (n; %)	4 (57.1)	12 (70.6)	11 (68.8)	15 (75.0	8 (61.5)	50 (68.5)
Chief (n; %)	1 (14.3)	1 (5.9)	3 (18.7)	1 (5.0)	2 (15.4)	8 (11.0)

### Exam results

**Fig. 2:**
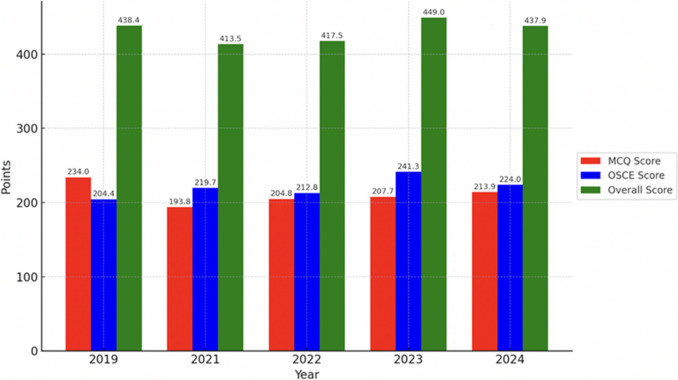
Exam results: multiple-choice test (MCQ), objective structured clinical examination OSCE, and overall scores (2019–2024). Both the MCQ and OSCE maximum possible score was 300 each making the maximum possible exam score 600

Overall exam pass rates, as well as performance in the MCQ and OSCE components, showed variation between years (Fig. 2). In 2019, the pass rate was 88.2% (15 of 17 candidates), with a mean MCQ score of 234.0 ± 17.1 points (78.0%), an OSCE score of 204.4 ± 26.9 points (68.1%), and an overall average of 438.4 ± 31.4 points (73.1%). During the 2021 and 2022 examinations, pass rates were lower compared to other years, with 19 of 31 (61.3%) and 14 of 20 (70.0%) passing in 2021 and 2022, respectively. MCQ scores averaged 193.8 ± 19 points (64.6%) in 2021 and 204.8 ± 29 points (68.3%) in 2022, while the OSCE results were 219.7 ± 32.4 points (73.2%) and 212.8 ± 42.8 points (70.9%). The overall mean scores for these two years were 413.5 ± 45.1 points (68.9%) in 2021 and 417.0 ± 60.9 points (69.6%) in 2022. In 2023, the pass rate increased to 77.3% (17/22), accompanied by an MCQ average of 207.7 ± 21.8 points (69.2%) and the highest recorded OSCE mean score of 241.3 ± 27.2 points (80.4%), resulting in an overall mean of 449.0 ± 42.9 points (74.8%). Finally, in 2024, the pass rate remained high at 81.3% (13/16), with an MCQ score of 213.9 ± 17.9 points (71.3%), an OSCE average of 224.0 ± 38.9 points (74.7%), and an overall mean score of 437.9 ± 43.3 points (73.0%).

### Correlation between credit scores and exam results

Participants’ logbook data were analyzed in relation to their examination results. This analysis was limited to the years 2021–2024 (*n* = 80), as in earlier years the logbooks were managed by another company rather than EAES, and the corresponding data were not available. Regression analyses showed no correlation between the number of laparoscopic operations performed and OSCE scores (Supp. Material Figure S3), nor between CME/Hands-on Training credits recorded in the logbook and MCQ scores (Supp. Material Figure S4). There was also no correlation between total logbook credit scores and total examination scores (Supp. Material Figure S5).

**Fig. 3:**
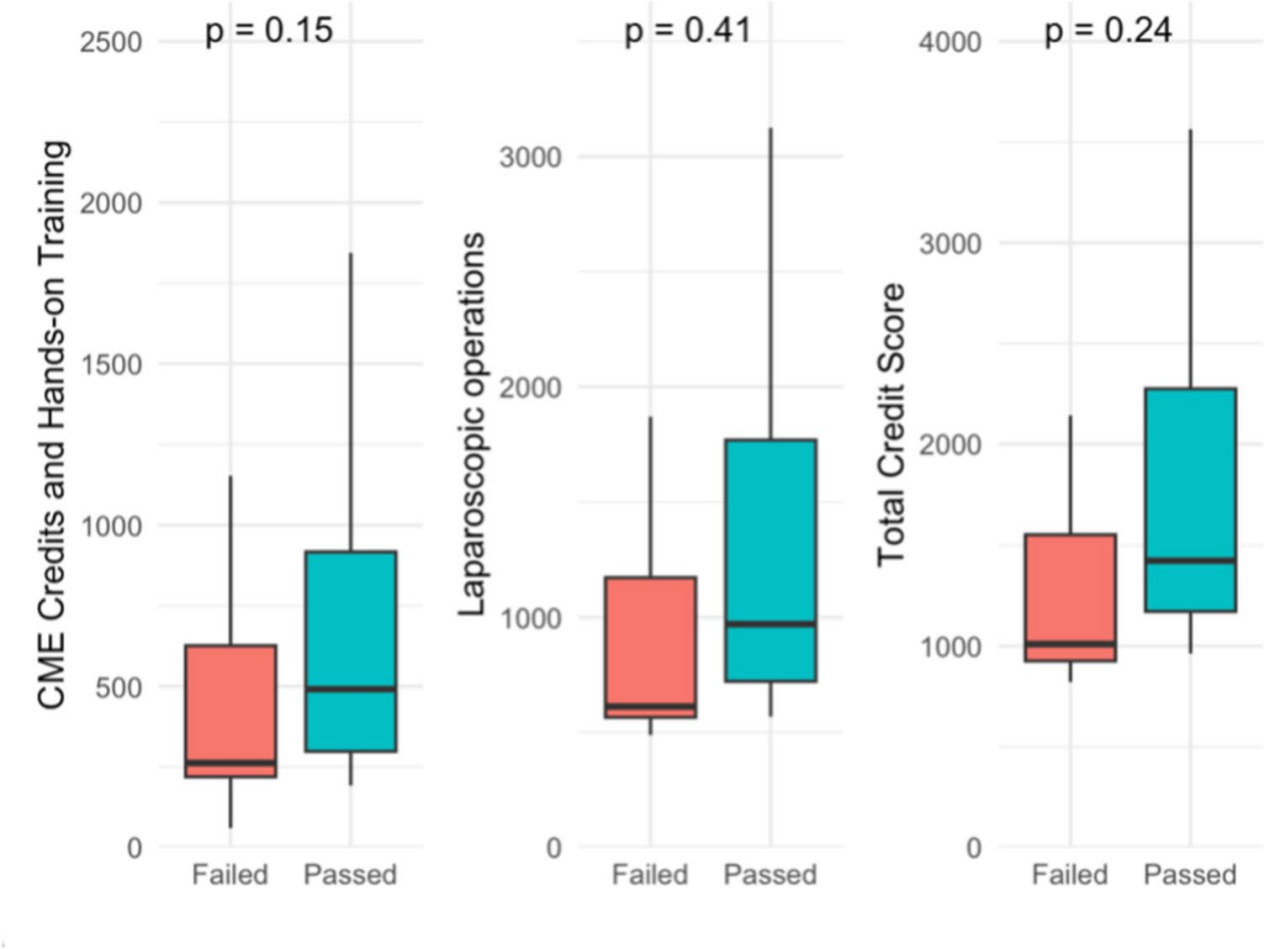
CME/Hands-on training credits, laparoscopic operations, and total credit score between participants who failed and passed

Participants who passed tended to have higher median CME/Hands-on Training credit scores, had performed more laparoscopic operations, and had higher total credit scores (Fig. 3*; *Table 3); however, these differences did not reach statistical significance, likely due to the limited sample size.

**Table 3:** CME/Hands-on training credits, laparoscopic operations, and total credit score between participants who failed and passed (2021–2024)

Logbook Categories	Failed (*n *= 20)	Passed (*n* = 60)	*p*-value
CME/Hands-on training credit points	262 (219,626)	514 (298,998)	0.15
Laparoscopic operations performed	612 (565,1172)	998 (726,1890)	0.41
Total credit score	1010 (918,1552)	1482 (1276,2330)	0.24

Data are presented as Median (Q1,Q3)

In summary, participants who passed the exam had higher credit scores, suggesting greater experience, than those who failed. However, the exam score was not directly correlated with the credit score, indicating that greater experience did not necessarily translate into better test performance.

### Optimal timing and motivations for taking the exam

51% reported their fellowship period was the best time to undertake the F.E.B.S./MIS. 38% believed the ideal time was as an attending surgeon, and 10% would have preferred taking it as specialists shortly after finishing their residency. Notably, no participants selected the role of chief as the best time to take the exam.

The primary motivations reported for taking the exam were to obtain an internationally recognized certificate (32%) and to update their knowledge (31%). These were followed by aspirations to enhance CVs (16%) and skills (11%) as to showcase expertise in MIS (10%) (Table 4).

**Table 4:** Self-reported motivation to take the exam

	2018	2019	2020–2022	2023	2024	Overall
n	8	23	17	20	20	88
Get an internationally accepted certificate (*n*; %)	3 (37.5)	7 (30.4)	7 (43.8)	7 (35.0)	4 (20.0)	28 (31.8)
Update knowledge (*n*; %)	3 (37.5)	7 (30.4)	4 (25.0)	7 (35.0)	6 (30.0)	27 (30.7)
Improve skills (*n*; %)	1 (12.5)	3 (13.0)	3 (18.8)	0 (0.0)	3 (15.0)	10 (11.4)
Improve CV (*n*; %)	1 (12.5)	6 (26.1)	3 (18.8)	3 (15.0)	1 (5.0)	14 (15.9)
Show MIS knowledge (*n*; %)	0 (0.0)	0 (0.0)	0 (0.0)	3 (15.0)	6 (30.0)	9 (10.2)

### Exam feedback

Participants primarily learned about the exam through the UEMS webpage (43%), followed by from colleagues (30%) and EAES mailings (18%). A smaller percentage found out through social media (6.8%) and other sources (2.7%) (Table 5*)*. These figures were static throughout the time period.

**Table 5:** Sources of exam awareness reported by participants

	2018	2019	2020–2022	2023	2024	Overall
n	6	17	16	21	13	73
Webpage (n; %)	1 (16.7)	7 (41.2)	9 (56.3)	9 (42.9)	5 (38.5)	31 (42.5)
Colleagues (n; %)	4 (66.7)	3 (17.6)	2 (12.5)	7 (33.3)	6 (46.2)	22 (30.1)
Social media (n; %)	2 (33.3)	1 (5.9)	1 (6.3)	0 (0.0)	1 (7.7)	5 (6.8)
EAES-mailing (n; %)	0 (0.0)	3 (17.6)	4 (25.0)	6 (28.6)	0 (0.0)	13 (17.8)
Others (n; %)	1 (16.7)	0 (0.0)	0 (0.0)	0 (0.0)	1 (7.7)	2 (2.7)

**Fig. 4:**
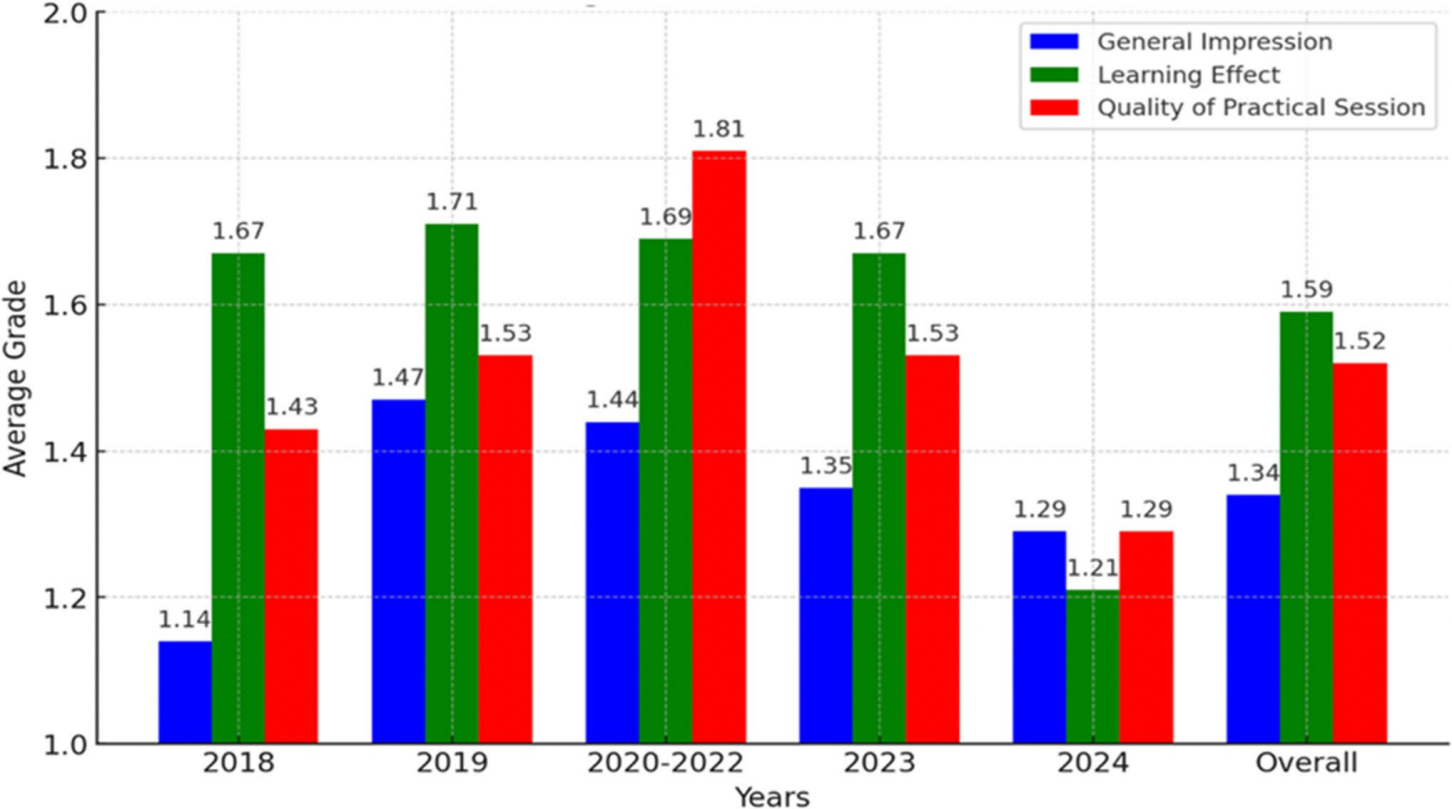
Trends in average grades for general impression, learning effect, and practical session quality. Participants used a Likert scale between 1 (best) and 5 (worst) for each component. Lower scores represent increasing satisfaction

Participants were also asked to grade several aspects of the F.E.B.S./MIS exam, including the general impression, the learning effect, and whether the practical exercises reflected the basic skills necessary for performing MIS. The general impression was highly positive with an average of 1.3 (Likert scale 1 (best) – 5 (worst)). The learning effect was graded with an average of 1.6, while the quality of the practical sessions was rated at 1.5, also reflecting strong satisfaction (Fig. 4). 2024 was the highest-scored exam. When asked about their preference for an open-book MCQ exam format, participants were evenly divided with a slight majority, 53% supporting this option.

**Fig. 5:**
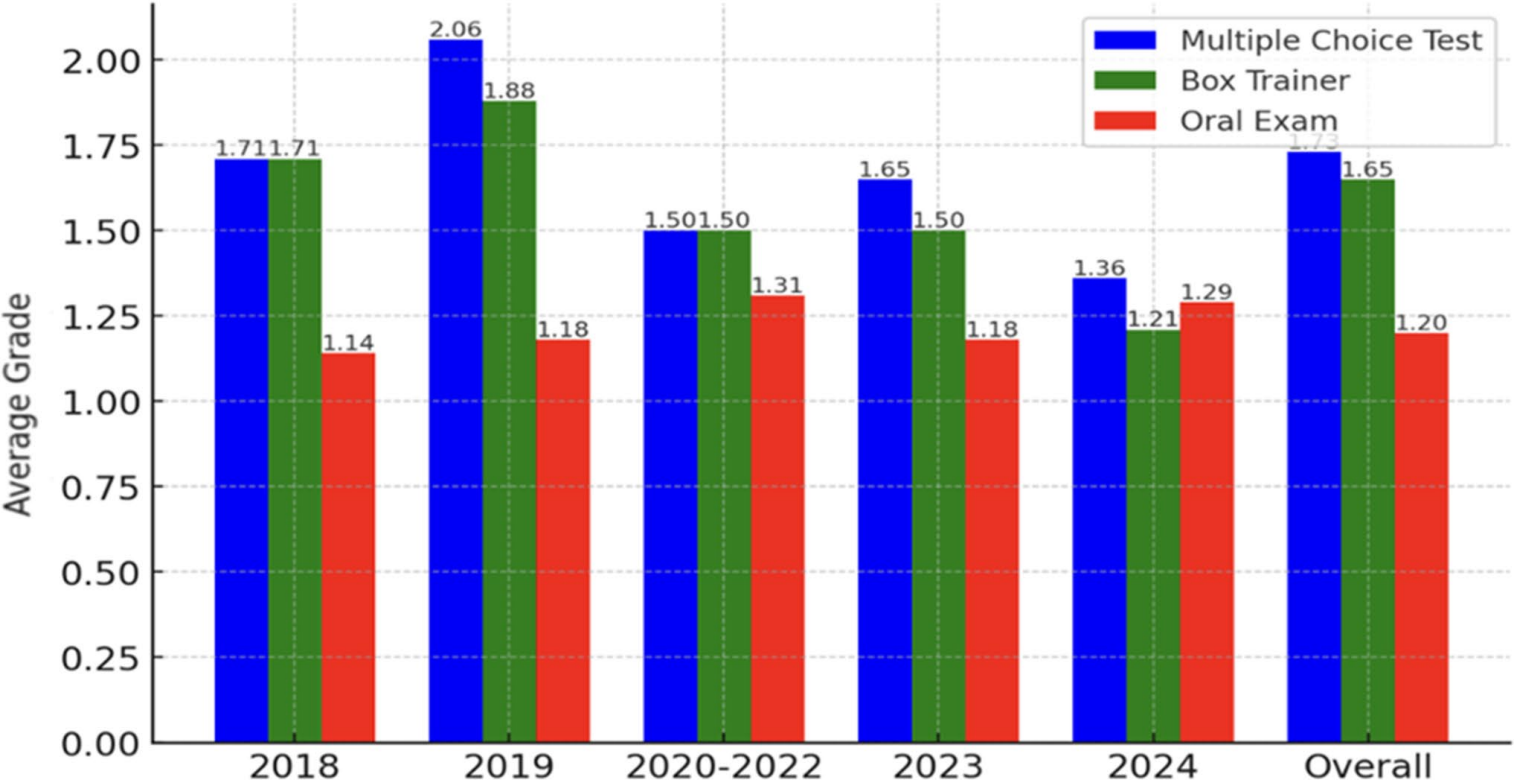
Trends in average participant grading for the MCQ test, skills assessment, and oral exam components. Participants used a Likert scale between 1 (best) and 5 (worst) for each component. Lower scores represent increasing satisfaction

The case-based oral exam was consistently rated as the most useful component for daily MIS practice with an average score of 1.2. The practical skills tests and MCQ tests both received an average score of 1.7 (Fig. 5). Scores were generally static across the time period.

Through free text and group sessions, responses from participants highlighted excellent exam organization, emphasized the opportunity to share experiences and practice prior to the exam as key strengths. Additionally, they appreciated the international board of examiners, recognizing the diversity it brought to the process. The broad spectrum of topics covered during the exam was also acknowledged, with many participants noting this as comprehensive. Overall, 94.2% of participants stated they would take the exam again, and 97.2% would recommend it to others. When asked about areas for improvement, many highlighted the application process, suggesting it could be streamlined or made more user-friendly. Others emphasized the need for a greater focus on practical components and additional opportunities to practice the stations just before the exam. Flexible test dates were also a common recommendation, as was the option to take the exam in their home country or primary language.

## Discussion

The F.E.B.S./MIS Board exam program, representing a collaboration of the UEMS and EAES, aimed to provide subspecialty MIS summative assessment not previously available and address identified gaps in the standardization and formal recognition of MIS expertise. Our initial experiences received supportive participant data and evidence of the interest and future viability of the program. The exam appears to successfully act as a benchmark specialist level MIS certification for international candidates. This supports the goals of the UEMS section of surgery to underpin the development of a highly skilled surgical workforce across Europe and work toward ensuring consistent training and skills validation across Europe and beyond [6, 7]. By improving on areas of feedback and building on its strengths, the program can assist in ensuring surgical standards, support professional growth, and assist the drive to better patient outcomes from high-quality MIS services.

This study highlights both the successes and the opportunities for further improvement of the program.

Participant demographics revealed a diverse pool of candidates from five continents underscoring the exam’s international appeal and the global interest in MIS accreditation. The eligibility threshold appears appropriate as participants had an average of 11.5 years of experience and had performed over 1200 MIS operations. One of the key findings of this study is the perceived suitability of the diploma during the fellowship or early attending surgeon stages. This highlights its role in facilitating the transition from training to independent practice. Structured certification programs provide a critical framework for bridging the gap between supervised learning and autonomous clinical practice, particularly in highly technical fields like MIS. The examination structure, including both theoretical and practical components for psychomotor and cognitive assessments covering all layers of Miller’s pyramid ensures candidates are broadly evaluated at high levels as appropriate for high stakes summative assessment of senior participants [8, 9]. Participant feedback confirmed the acceptability and face validity of the exam structure and content. This report should inform and reassure potential future candidates and enhance the transparency of the exam for participants and surgical assessors.

A question that arises is whether greater documented surgical experience translates into better examination performance. Interestingly, our analysis of logbook data from 2021 to 2024 suggests that it may not. Although candidates who passed tended to have higher credit scores and more recorded procedures, these differences did not reach statistical significance, likely due to the limited sample size. This indicates that examinations may assess broader competencies not fully captured by logbook records.

This observation is consistent with findings from other studies. For example, a study involving surgical residents and fellows in the United States and Canada found that while higher consumer credit scores were moderately associated with a greater likelihood of positive academic performance indicators—including top written exam scores, research productivity, and awards—the discriminatory ability of credit score alone was only moderate (AUC = 0.70), with limited specificity. Notably, low credit scores did not reliably predict negative outcomes, and the authors discouraged the use of such scores as a selection tool due to socioeconomic confounders and the complexity of surgical performance [10].

Similarly, a study of 184 medical students’ logbooks found no significant correlation between the number of clinical encounters (experience logged) and performance on either objective structured clinical examinations (OSCEs) or multiple-choice exams [11].

Together, these findings suggest that while experience and engagement are important, examination performance is influenced by additional factors such as cognitive skills, focused preparation, and adaptability—competencies not easily quantified through logbooks or credit scores.

Another broader question arising from this study is whether participating in certification programs like F.E.B.S./MIS contributes to improved surgical outcomes. While links between certification and clinical performance are multifactorial and challenging to investigate, evidence suggests that structured assessment frameworks promote lifelong learning and adherence to best practices [12, 13]. Surgeons who undergo certification processes are more likely to stay updated with evolving techniques and demonstrate higher levels of technical competence [14] supporting the view that exams are not merely academic exercises or resumé enhancing but integral components of continuing professional development [15].

The successful delivery of this examination requires close collaboration between the EAES and the UEMS. As the exam is jointly conducted under the authority of both bodies, significant coordination is needed among organizational leaders, the examination board, and candidates. Over the past seven years, logistical challenges—particularly in communication, candidate correspondence, and marketing—have been identified and progressively addressed. These changes have led to a more efficient administrative process and a consistently improved candidate experience, as reflected in annual feedback.

Despite the positive evaluations, several actionable areas were identified for ongoing improvement. The application process can be streamlined and a longer application window could be provided. There was also a call for more emphasis on practical components and additional opportunities to practice before the exam. Flexible test dates and locations were a common request which could improve accessibility and reduce logistical challenges and participant expenses. Nevertheless, such an approach would likely increase the financial burden for the exam organization. The split opinion on the preference for an open-book MCQ exam is interesting. Open book tests promote assessment of critical thinking over the traditional fact recall and basic interpretation of information and may be a more appropriate tool for summative assessment at the specialist level. This was carefully considered by the working group. In 2025 a robotic specific exam was piloted in Belgrade in response to MIS evolution.

To ensure the quality and sustainability of the exam, the EAES/UEMS MIS Working Group convenes regularly to review candidate evaluations and operational outcomes. These meetings serve as a platform for ongoing quality assurance, enabling the group to identify recurrent issues, implement refinements, and streamline logistics year over year. This structured, feedback-driven approach ensures the exam remains not only technically rigorous but also logistically sound and responsive to the needs of candidates and examiners alike.

The overwhelmingly positive feedback, including a 94% willingness to retake the exam and a 97% recommendation rate, attests to the program’s success in meeting participants’ expectations. Participants’ emphasis on the program’s organization and the international expertise of examiners reflects the high standard maintained by the EAES and UEMS in ensuring a robust certification process. Following successful establishment, the program has significant potential for further growth. Expanding accessibility to more regions and offering tailored preparation resources could attract a wider range and higher number of participants. Additionally, using feedback to refine the practical and theoretical components will ensure that the diploma continues to meet the continuously evolving needs of MIS practitioners.

Limitation of this study include not all participants completing the evaluation questionnaire which may introduce a degree of selection bias in the findings and risks limiting the generalizability of the data. No formal examiner feedback nor comment from the UEMS and EAES was collated for review. Additionally, credit score data were only available for the period 2021–2023, which restricts longitudinal analysis and may limit the comprehensiveness of the findings.

## Conclusion

The F.E.B.S./MIS examination is positioned as an important credential for the international accreditation of MIS specialists; however, its actual impact and degree of recognition warrant further systematic evaluation.

## Supplementary Information

Below is the link to the electronic supplementary material.

Supplementary file1 (PDF 202 KB)

Supplementary file2 (PDF 201 KB)

Supplementary file3 (DOCX 17 KB)

Supplementary file4 (PNG 122 KB)

Supplementary file5 (PNG 115 KB)

Supplementary file6 (PNG 62 KB)
