# Supplementary material for: Fellowship of the European Board of Surgery in the specialty of Minimally Invasive Surgery (F.E.B.S./MIS): a continuous evaluation
Source: Surg Endosc. 2025 Sep 19;39(11):7103–13. doi: 10.1007/s00464-025-12204-3 (PMC12618417; doi:10.1007/s00464-025-12204-3)
Supplement: Supplementary file 1 — Supplementary file1 (PDF 202 KB) [file 464_2025_12204_MOESM1_ESM.pdf]

# LOGBOOK EBSQ/MIS for Applicants Performing Endoscopy

**Please refer to the Catalogue of Procedures & Operations for description of categories**
**Field "Principal surgeon" = fill in NUMBER of procedures performed as operating surgoen**
**Field "Assistant surgeon" = fill in NUMBER of procedures performed as assisting surgeon**

**Name and Surname of applicant:**
**Hospital:**

| Category A: Endoscopies (minumum 200 credit points) | Principal surgeon | Assistant surgeon | Credit points |
|---|---|---|---|
| **A1 - Flexible EGD (minimum 20 as principal surgeon)** | | | |
| **A2 - Flexible colonoscopy (minimum 25 as principal surgeon)** | | | |
| **A3 - Endoscopic interventions (minimum 5 as principal surgeon)** | | | |
| **Total credit points Category A (minimum 200):** | | | |

| Category B: Basic Laparoscopic Operations (minimum 300 credit points) | Principal surgeon | | Credit points |
|---|---|---|---|
| **B1 - Appendectomy (minimum 19 as principal surgeon)** | | | |
| **B2 - Cholecystectomy (minimum 75 as principal surgeon)** | | | |
| **B3 - Diagnostic/ therapeutic laparoscopy (minimum 19 as principal surgeon)** | | | |
| **Total credit points Category B (minimum 300):** | | | |

| Category C: Advanced Laparoscopic Operations (300 credit points) | Principal surgeon | Assistant surgeon | Credit points |
|---|---|---|---|
| **Minimum 75 as principal surgeon** | | | |
| **Minimum 2 subcategories with at least 10 procedures each as principal surgeon** | | | |
| **C1 - Abdominal wall hernia repair** | | | |
| **C2 - Hiatal hernia repair, antireflux procedures** | | | |
| **C3 - Bariatric procedures** | | | |
| **C4 - Gastric/duodenal/small bowel resection** | | | |
| **C5 - HPB** | | | |

| | Number completed | Points per activity | Credit points |
|---|---|---|---|
| **C6 - adrenalectomy, splenectomy** | | | |
| **C7 - Colon & Rectum** | | | |
| **C8 - Transanal (TAMIS, TEM)** | | | |
| **C9 - Transoral (POEM, Zenker diverticulectomy)** | | | |
| **C10 - Thoracic (VATS) procedures** | | | |
| **Total credit points Category C (minimum 300):** | | | |

| Category D: CME Credits and Hands-on Training (200 credit points) | Number completed | Points per activity | Credit points |
|---|---|---|---|
| **D1 - CME credits (minimum 50 credit points)** | | | |
| Participation at national congress related to minimally invasive surgery (MIS) | | 4 | |
| Poster presentation at national congress (first author, MIS related) | | 6 | |
| Oral presentation at national congress (presenting author, MIS related) | | 8 | |
| Participation at recognized international congress related to MIS (e.g. EAES, SAGES, WCES) | | 8 | |
| Poster presentation at recognized internatiional congress (first author, MIS related) | | 12 | |
| Oral presentation at recognized international congress (presenting author, MIS related) | | 16 | |
| Participation at recognized theoretical postgraduate course (MIS related) | | 12 | |
| MIS related publication in national peer-reviewed journal (first/corresponding author) | | 12 | |
| MIS related publication in international peer-reviewed journal (first/corresponding author) | | 24 | |
| **D2 - Credits for hands-on training (minimum 150 credit points)** | | | |
| Basic laparoscopy course - refer to catalogue (certificate required) | | 30 | |
| Advanced (procedure/organ/pathology specific) course - (certificate required) | | 60 | |
| Fellowship/clinical stay at recognized MIS center - (certificate required) | | 10/week | |
| Faculty/trainer at recognized MIS course/center - (certificate required) | | 60 | |
| **Total credit points Category D (minimum 200):** | | | |

| TOTAL CREDIT POINTS Category A+B+C+D (minimum 1000): | |
|---|---|

**Date & place:**

**Name, Surname, Signature and Stamp of Supervisor:**
