# Supplementary material for: Fellowship of the European Board of Surgery in the specialty of Minimally Invasive Surgery (F.E.B.S./MIS): a continuous evaluation
Source: Surg Endosc. 2025 Sep 19;39(11):7103–13. doi: 10.1007/s00464-025-12204-3 (PMC12618417; doi:10.1007/s00464-025-12204-3)
Supplement: Supplementary file 2 — Supplementary file2 (PDF 201 KB) [file 464_2025_12204_MOESM2_ESM.pdf]

LOGBOOK EBSQ/MIS for Applicants NOT Performing Endoscopy

Please refer to the Catalogue of Procedures & Operations for description of categories
Field "Principal surgeon" = fill in NUMBER of procedures performed as operating surgoen
Field "Assistant surgeon" = fill in NUMBER of procedures performed as assisting surgeon
Missing 200 credit points for Category A - Endoscopies must be added to Category B and/or C, so category B+C= minimum 800 points

Name and Surname of applicant:
Hospital:

Category B: Basic Laparoscopic Operations (minimum 300 credit points)	Principal surgeon		Credit points
**B1 - Appendectomy (minimum 19 as principal surgeon)**			
**B2 - Cholecystectomy (minimum 75 as principal surgeon)**			
**B3 - Diagnostic/ therapeutic laparoscopy (minimum 19 as principal surgeon)**			
**Total credit points Category B (minimum 300):**			

Category C: Advanced Laparoscopic Operations (300 credit points)	Principal surgeon	Assistant surgeon	Credit points
**Minimum 75 as principal surgeon**			
**Minimum 2 subcategories with at least 10 procedures each as principal surgeon**			
**C1 - Abdominal wall hernia repair**			
**C2 - Hiatal hernia repair, antireflux procedures**			
**C3 - Bariatric procedures**			
**C4 - Gastric/duodenal/small bowel resection**			
**C5 - HPB**			
**C6 - adrenalectomy, splenectomy**			
**C7 - Colon & Rectum**			
**C8 - Transanal (TAMIS, TEM)**			
**C9 - Transoral (POEM, Zenker diverticulectomy)**			
**C10 - Thoracic (VATS) procedures**			
**Total credit points Category C (minimum 300):**			

Total credit points Category B+C (minimum 800):			

Category D: CME Credits and Hands-on Training (200 credit points)	Number completed	Points per activity	Credit points
**D1 - CME credits (minimum 50 credit points)**			
Participation at national congress related to minimally invasive surgery (MIS)		4	
Poster presentation at national congress (first author, MIS related)		6	
Oral presentation at national congress (presenting author, MIS related)		8	
Participation at recognized international congress related to MIS (e.g. EAES, SAGES, WCES)		8	
Poster presentation at recognized internatiional congress (first author, MIS related)		12	
Oral presentation at recognized international congress (presenting author, MIS related)		16	
Participation at recognized theoretical postgraduate course (MIS related)		12	
MIS related publication in national peer-reviewed journal (first/corresponding author)		12	
MIS related publication in international peer-reviewed journal (first/corresponding author)		24	
**D2 - Credits for hands-on training (minimum 150 credit points)**			
Basic laparoscopy course - refer to catalogue (certificate required)		30	
Advanced (procedure/organ/pathology specific) course - (certificate required)		60	
Fellowship/clinical stay at recognized MIS center - (certificate required)		10/week	
Faculty/trainer at recognized MIS course/center - (certificate required)		60	
**Total credit points Category D (minimum 200):**			

**TOTAL CREDIT POINTS Category A+B+C+D (minimum 1000):**			

Date & place:

Name, Surname, Signature and Stamp of Supervisor:
