# Supplementary material for: Fellowship of the European Board of Surgery in the specialty of Minimally Invasive Surgery (F.E.B.S./MIS): a continuous evaluation
Source: Surg Endosc. 2025 Sep 19;39(11):7103–13. doi: 10.1007/s00464-025-12204-3 (PMC12618417; doi:10.1007/s00464-025-12204-3)
Supplement: Supplementary file 3 — Supplementary file3 (DOCX 17 KB) [file 464_2025_12204_MOESM3_ESM.docx]

Supplementary material (Figure S1)

**Evaluation of F.E.B.S./MIS examination**

**1. How did you find out about the F.E.B.S./MIS exam?**

___ Webpage of UEMS ___ Colleagues

___ Social media (Facebook, ...) ___ EAES-Mailing

others: ________________

**2. Would you please describe shortly why did you take the F.E.B.S./MIS exam? What was your motivation?**

___________________________________________________________________

___________________________________________________________________

**3. Please, let us know your clinical experience.**

Total years of surgical practice: _________________

Total number of minimally invasive surgeries performed: _____________

Your current job position (please circle):
 Specialist Fellow Attending/Consultant Chief

**4. What stage of career do you think is best to take this exam? Please circle.**

Specialist Fellow Attending/Consultant Chief

**5. Please rate the statements 1 to 3 on a scale of 1 (**very good) **to 5** (very bad)

| **Statement** | 1-5 |
| --- | --- |
| 1. How was your general impression of the F.E.B.S./MIS exam? |  |
| 2. Characterize the learning effect of the F.E.B.S./MIS exam? |  |
| 3. Did practical exercises of the F.E.B.S./MIS exam reflect the basic   skills necessary for performance of minimally invasive surgery? |  |
| 4. Would you take the F.E.B.S./MIS exam again? | Yes / No |
| 5. Would you recommend to other colleagues the F.E.B.S./MIS exam? | Yes / No |

**6. Please rate the usefulness of the examination parts for daily practice in MIS on a scale of 1 (**very useful) **to** **5 (**not useful at all)**:**

| **Exercise** | 1-5 |
| --- | --- |
| Multiple choice knowledge tests |  |
| Practical skills tests on Box-trainers |  |
| Case-based oral exam |  |

**7. What would you recommend for improvement of the F.E.B.S./MIS exam?**

___________________________________________________________________

___________________________________________________________________

**8. What was particular good regarding the F.E.B.S./MIS exam?**

___________________________________________________________________

___________________________________________________________________

**9. Would you prefer to do the multiple-choice test as an "open book" exam? That means sitting the test at any time at your home computer without supervision.**

Yes No
