# Supplementary figures and images for: Fellowship of the European Board of Surgery in the specialty of Minimally Invasive Surgery (F.E.B.S./MIS): a continuous evaluation
Source: Surg Endosc. 2025 Sep 19;39(11):7103–13. doi: 10.1007/s00464-025-12204-3 (PMC12618417; doi:10.1007/s00464-025-12204-3)

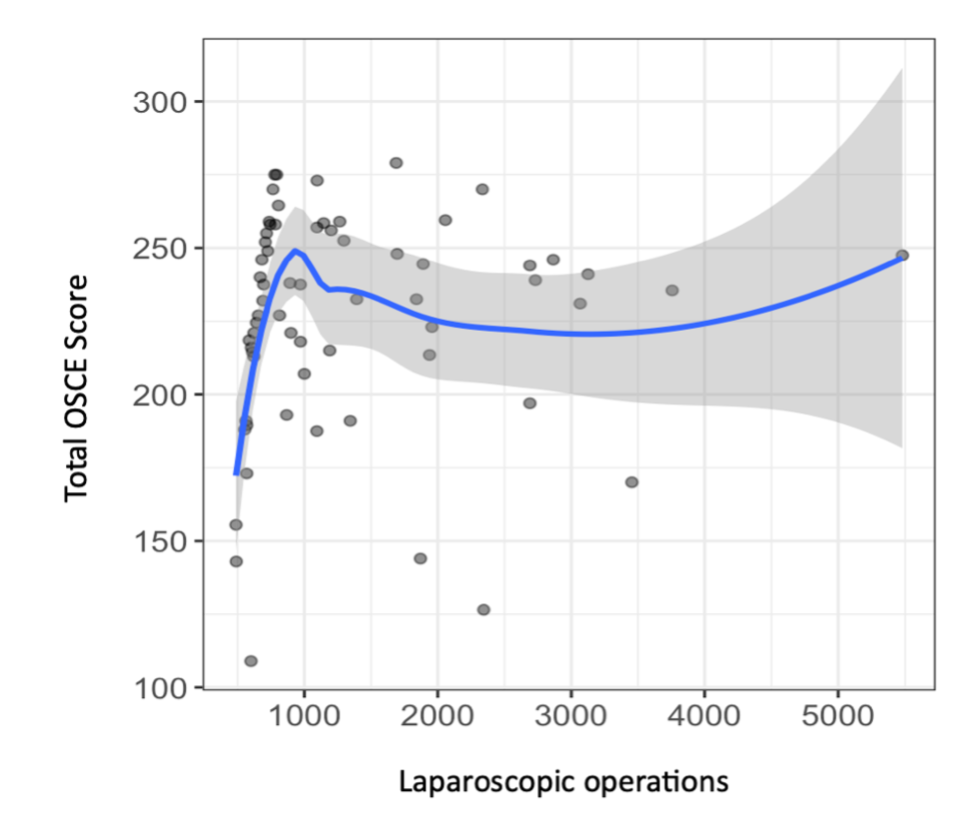

Supplement: Supplementary file 4 — Supplementary file4 (PNG 122 KB) [file 464_2025_12204_MOESM4_ESM.png]

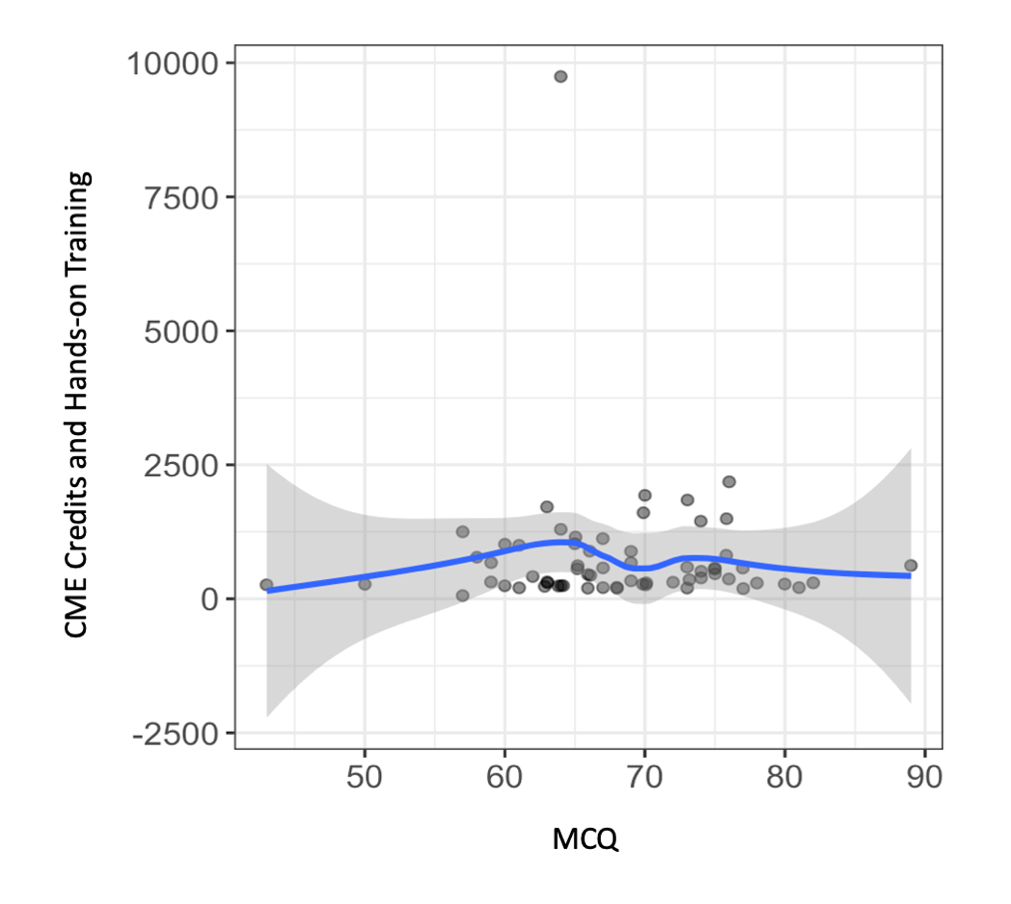

Supplement: Supplementary file 5 — Supplementary file5 (PNG 115 KB) [file 464_2025_12204_MOESM5_ESM.png]

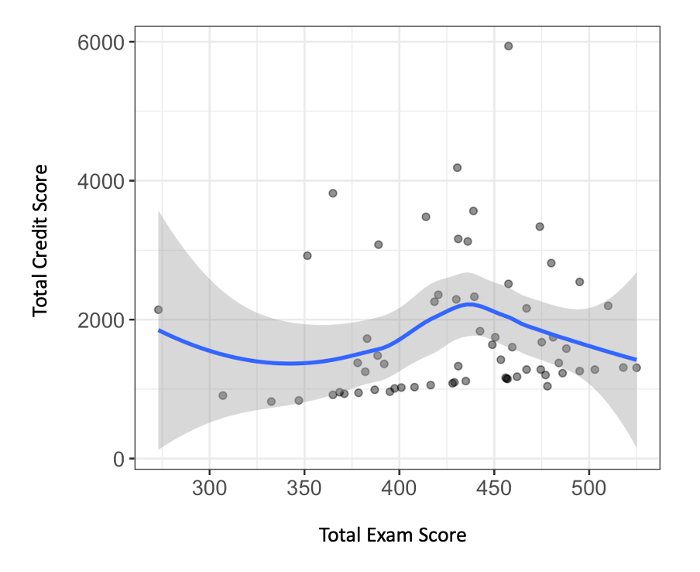

Supplement: Supplementary file 6 — Supplementary file6 (PNG 62 KB) [file 464_2025_12204_MOESM6_ESM.png]
